# Red blood cell transfusions worsen the outcomes even in critically ill patients undergoing a restrictive transfusion strategy

**DOI:** 10.1590/S1516-31802012000200002

**Published:** 2012-04-03

**Authors:** João Manoel da Silva, Ederlon Rezende, Cristina Prada Amendola, Rafael Tomita, Daniele Torres, Maria Tereza Ferrari, Diogo Oliveira Toledo, Amanda Maria Ribas Rosa Oliveira, Juliana Andreia Marques

**Affiliations:** I MD, MSc. Physician, Intensive Care Department, Hospital do Servidor Público Estadual (HSPE), São Paulo, Brazil.; II MD. Physician, Intensive Care Department, Hospital do Servidor Público Estadual (HSPE), São Paulo, Brazil.; III MD. Physician, Intensive Care Department, Hospital de Câncer de Barretos, Fundação Pio XII, Barretos, São Paulo, Brazil.; IV MD. Resident, Intensive Care Department, Hospital do Servidor Público Estadual (HSPE), São Paulo, Brazil.

**Keywords:** Anemia, Blood transfusion, Intensive care, Hospital mortality, Morbidity, Anemia, Transfusão de sangue, Cuidados intensivos, Mortalidade hospitalar, Morbidade

## Abstract

**CONTEXT AND OBJECTIVE:**

Anemia and blood transfusions are common in intensive care. This study aimed to evaluate epidemiology and outcomes among critically ill patients under a restrictive transfusion strategy.

**DESIGN AND SETTING:**

Prospective observational cohort study in an intensive care unit (ICU) at a tertiary hospital.

**METHODS:**

All adults admitted to the ICU over a one-year period who remained there for more than 72 hours were included, except those with acute coronary syndrome, ischemic stroke, acute hemorrhage, prior transfusion, pregnant women and Jehovah’s Witnesses. The restrictive strategy consisted of transfusion indicated when hemoglobin levels were less than or equal to 7.0 g/dl.

**RESULTS:**

The study enrolled 167 patients; the acute physiology and chronic health evaluation II (APACHE II) score was 28.9 ± 6.5. The baseline hemoglobin level was 10.6 ± 2.2 g/dl and on day 28, it was 8.2 ± 1.3 g/dl (P < 0.001). Transfusions were administered to 35% of the patients. In the transfusion group, 61.1% did not survive, versus 48.6% in the non-transfusion group (P = 0.03). Transfusion was an independent risk factor for mortality (P = 0.011; odds ratio, OR = 2.67; 95% confidence interval, CI = 1.25 to 5.69). ICU stay and hospital stay were longer in the transfusion group: 20.0 (3.0-83.0) versus 8.0 (3.0-63.0) days (P < 0,001); and 24.0 (3.0-140.0) versus 14.0 (3.0-80.0) days (P = 0.002), respectively.

**CONCLUSIONS:**

In critically ill patients, there was a reduction in hemoglobin with increasing length of ICU stay. Moreover, transfusion was associated with worse prognoses.

## RESUMO

**CONTEXTO E OBJETIVO:**

Anemia e transfusões sanguíneas são comuns em terapia intensiva. O objetivo deste estudo foi investigar a epidemiologia e resultados em pacientes críticos sob o regime de transfusão restritiva.

**TIPO DE ESTUDO E LOCAL:**

Estudo de coorte, prospectivo e observacional em unidade de terapia intensiva de um hospital terciário.

**MÉTODOS:**

Foram incluídos todos adultos admitidos na unidade durante um ano e que permaneceram internados por mais de 72 horas, exceto pacientes com lesão coronariana aguda, isquemia cerebral aguda, hemorragia aguda, transfusão anterior, mulheres grávidas e testemunhas de Jeová. A estratégia restritiva consiste na transfusão indicada com a hemoglobina inferior ou igual a 7.0 g/dl.

**RESULTADOS:**

Totalizando 167 pacientes incluídos, APACHE II (Acute Physiology and Chronic Health disease II) foi de 28,9 *±* 6,5. O valor da hemoglobina basal foi de 10,6 *±* 2.2 g/dl e no 28^o^ dia foi de 8,2 *±* 1.3 g/dl (P < 0,001). 35% dos pacientes receberam transfusões. No grupo de transfusão 61,1% não sobreviveram *versus* 48,6% do grupo não transfusão (P = 0,03). A transfusão foi fator de risco independente de mortalidade (P = 0,011; *odds ratio*, OR = 2,67; intervalo de confiança, IC 95% = 1,25-5,69). A internação na unidade de terapia intensiva e hospitalar foi maior no grupo de transfusão: 20,0 (3,0-83,0) *versus* 8,0 (3,0-63,0) dias (P < 0,001); e 24,0 (3,0-140,0) *versus* 14,0 (3,0-80,0) dias (P = 0,002).

**CONCLUSÕES:**

Em pacientes graves, observou-se redução da hemoglobina com a progressão da permanência na UTI. Além disso, a transfusão foi associada com piores prognósticos.

## INTRODUCTION

Anemia is a common condition in critically ill patients. Although a few patients admitted to intensive care units have normal hemoglobin levels on admission, nearly all patients become anemic over the course of their intensive care unit stays.^1^ Almost 95% of patients admitted to intensive care units have hemoglobin levels that are below normal, especially after three days in the intensive care unit.^2^

The cause of this anemia is likely to be multifactorial.^3^ The anemia is associated with high morbidity and mortality rates in some groups of patients, probably secondary to tissue hypoxia.^3^

Nowadays, blood transfusion plays a pivotal role in managing acute anemia in intensive care patients, with the aims of reducing tissue hypoxia and increasing the oxygen supply to tissues and organs.^4^ A recent report showed that 85% of patients staying in intensive care for one week underwent blood transfusions.^5^

However, this treatment is not free from side effects. Patients undergoing transfusion have higher mortality rates in the intensive care unit and hospital, higher rates of organ dysfunction and longer stays in intensive care units.^6^^,^^7^ Hébert et al. conducted a randomized multicenter study and demonstrated that a restrictive transfusion strategy (transfusion implemented when hemoglobin rates are lower than 7.0 g/dl) is safe and effective. Moreover, they showed that there were better outcomes from a restrictive strategy among patients with less severe illness (acute physiology and chronic health evaluation II score < 20) and younger patients (< 55 years).^8^ In addition, another study showed that individuals with euvolemic anemia and hemoglobin levels between 3.5 and 5 g/dl did not develop organ dysfunction.^9^

Therefore, it is unclear whether there is any benefit for critically ill patients from a restrictive transfusion strategy in which blood transfusion indications are based uniquely on occurrences of hemoglobin levels below 7.0 g/dl.

## OBJECTIVE

This study had the aim of investigating epidemiology and outcomes relating to blood transfusion among critically ill patients who were all under a restrictive transfusion regime and who had remained in an intensive care unit for more than three days.

## METHODS

This prospective observational cohort study was conducted in a 20-bed medical-surgical intensive care unit in a tertiary-level hospital. All patients aged over 18 years old who were admitted to the intensive care unit between November 1, 2005, and November 1, 2006, and who remained there for more than three days were included. These patients were asked to sign an informed consent statement, agreeing to their participation in this study. Patients with acute hemorrhage, histories of previous transfusions, pregnant women, acute coronary disease or stroke were excluded, as were Jehovah’s Witnesses. Patients were followed up for 28 days after the blood transfusion or until hospital discharge or death, if it occurred before the end of this 28-day follow-up.

We evaluated demographic data, the acute physiology and chronic health evaluation II (APACHE II) score,^10^ the sequential organ failure assessment (SOFA),^11^ and the multiple organ dysfunction score (MODS)^12^ on admission, as part of the institution’s protocol for obtaining data on all hospitalized patients. Data gathering took place after the protocol for this study had been approved by the institution’s research ethics committee. The active search for patients and the data gathering were conducted by physicians who had been specially trained to control for possible mistakes.

The transfusion trigger and the decision on how many units of red blood cells to use were taken from the previous hemoglobin value that had been transfused. The hemoglobin level was recorded every day over the course of the length of intensive care unit stay. The tissue hypoxia markers used were arterial lactate, base differences, central venous oxygen saturation, diuresis and the difference of central venous CO_2_ minus arterial CO_2_. Occurrences of tissue hypoperfusion were defined as situations in which two of these markers presented abnormal values.

The researchers had no influence on the treatment administered to the patients. The blood transfusion protocol of this intensive care unit is that transfusion is only given to patients with hemoglobin levels lower than 7.0 g/dl. The exception is for patients with cardiovascular diseases and others in a state of tissue hypoperfusion associated with circulatory shock that requires catecholamine, for whom the hemoglobin level is kept around 7.0 to 9.0 g/dl.

### Statistical analysis

To determine the relative hospital death risk, we developed a multivariable analysis model for the population. Variables considered for the logistic regression analysis were introduced into this model if significantly associated with a higher risk of in-hospital death on a univariate basis at a P value of less than 0.2 or if they were clinically relevant variables.

The patients were divided in two groups (transfusion group and non-transfusion group) and they were compared in relation to demographic, clinical and laboratory variables.

Statistical analyses from means were compared between groups using the Student t test. For variables without normal distribution the Mann-Whitney test and ordinal variables were used. Estimates of hospital length of stay curves were calculated using the Kaplan-Meier method and their differences were tested using the scoring logarithm (log rank test). These variables were represented by the median and interquartile range. Categorical variables were analyzed using the chi-square test.

All significance probabilities (P value) presented were two-sided and values less than 0.05 were considered statistically significant. Odds ratios and their respective 95% confidence intervals were estimated through logistical regression. The data were shown as mean ? standard deviation, median (with interquartile range) or percentages. The statistical analysis was performed using the Statistical Package for the Social Sciences (SPSS) 13.0.

## RESULTS

Nine hundred and thirty-eight patients were admitted to intensive care unit over the study period. The following were excluded from this total: 484 patients with intensive care unit length of stay less than 72 hours or death before this time; 143 with blood transfusions before admission to the intensive unit care; 61 with acute hemorrhage; 48 with acute coronary syndrome; 32 with ischemic stroke; two patients who were pregnant; and one patient who was a Jehovah’s witness. Therefore, 167 patients who met the inclusion criteria were enrolled in the study; 44.3% were patients from wards and 39.5% were postoperative patients. Regarding previous illnesses, 55.4% had cardiovascular disease and 10.8% had no comorbidities. The mean age was 66.7 ? 13.8 years, and 58.7% were male. The mean APACHE II, SOFA and multiple organ dysfunction score were respectively 28.9 ? 6.5, 6.3 ? 2.8 and 7.9 ? 3.0. The hospital mortality rate was 54.8%.

**Figure 1.**
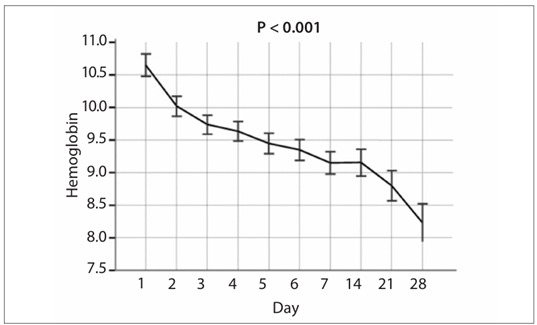
Hemoglobin trend for all patients over the 28-day period after transfusion.

Among the patients included, 35.3% received blood transfusion, with an average amount of two units (range: 1-3) of red blood cells. The median hemoglobin level before transfusion was 6.6 g/dl (range: 6.1 to 6.9). On admission to the intensive care unit, among all the patients in this study, the mean hemoglobin level was 10.6 ? 2.2 g/dl. After 28 days, this became 8.2 ? 1.3 g/dl (P < 0.001) (Figure 1).

Regarding the transfusion criteria, 77.6% of the cases received packed red blood cell transfusions because their hemoglobin levels were below 7 g/dl and 19.0% because of tissue hypoperfusion. Furthermore, patients who received blood transfusions because of hypoperfusion had a lower mortality rate than observed among the patients who received blood transfusions because their hemoglobin levels were below 7.0 g/dl (P = 0.001; 18.2% versus 65 9%) (Table 1).

In the univariate analysis between survivors and non-survivors in hospital, we found that female patients, individuals with high baseline SOFA or MODS, those originating from the emergency department or ward, cases with preexisting illnesses and those who received transfusions had a higher risk of mortality (P < 0.2) (Table 2). Thus, the variables with a higher risk of mortality in the univariate analysis were input to multivariate analysis in order to avoid confounding factors. Only red blood cell transfusion, female gender, baseline multiple organ dysfunction score, cardiovascular diseases and immunosuppressive diseases were independent risk factors for death. The SOFA was withdrawn from this analysis because the multiple organ dysfunction score^12^ has the same role and showed greater statistical significance than did the SOFA, in univariate analysis (Table 3).

**Figure 2.**
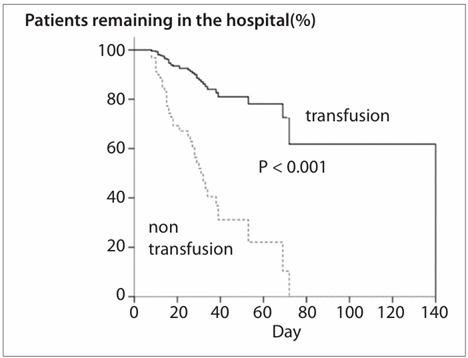
Hospital length of stay among non-transfusion and transfused patients.

The comparison between patients who received transfusion and those who did not showed that there were no statistical differences between the groups in relation to age, gender, APACHE II score, SOFA, baseline multiple organ dysfunction score, underlying disease and other factors. However, the group that received packed red blood cell transfusions had higher hospital and intensive care unit mortality rates, greater anemia and longer hospital and intensive care unit lengths of stay, as evaluated using the Kaplan-Meier method (Figure 2). All of these outcomes were statistically significant (Table 4).

**Figure 3.**
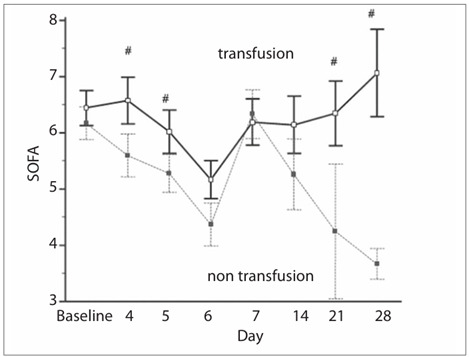
Sequential organ failure assessment (SOFA) trends among patients who received transfusions and those who did not receive transfusions.

Furthermore, the patients in the transfusion group showed no improvement in SOFA score on day 28 compared with the SOFA score on admission (6.4 ? 2.4 versus 7.1 ? 3.2; P = 0.34), while the group that did not receive transfusions showed a statistically significant improvement in SOFA score on day 28, compared with the baseline SOFA score (6.1 ? 2.9 versus 4.0 ? 0.8; P = 0.04) (Figure 3).

**Table 1.** Characteristics of patients included in the study, at the time of transfusion

Blood transfusion criteria	Characteristics
Hemoglobin < 7.0 g/dl	77%
Tissue hypoperfusion	19%
Cardiovascular disease	2%
Acute hemorrhage	2%
Hemoglobin level	6.6 (6.1-6.9) (g/dl)

For the hemoglobin level, the characteristics are expressed as the median (with 25-75 percentiles).

**Table 2.** Comparison amongst survivors and non-survivors

Variables	Non-survivors (n = 91)	Survivors (n = 76)	P
Age	66.6 ? 14.3	66.9 ? 13.4	0.87
Male (%)	51.6	68.0	0.03
Female (%)	48.4	32.0	
APACHE II	28.6 ? 7.1	29.3 ? 5.7	0.49
Baseline SOFA	6.6 ? 2.7	5.7 ? 2.8	0.04
Baseline MODS	7.2 ? 2.0	6.2 ? 1.8	0.01
Daily hemoglobin total (g/dl)	9.3 ? 1.6	9.5 ? 1.7	0.43
Origin (%)			
Ward	48.4	40.0	0.11
Surgery	33.0	48.0	
Emergency	18.7	10.7	
Other hospital	0.0	1.3	
Previous disease (%)			
Cardiovascular	63.3	45.3	0.11
Renal	8.9	14.7	
Immunodeficient	11.1	9.3	
Respiratory	8.9	10.7	
Hepatic	2.2	2.7	
Absent	5.6	17.3	
Ventilation mode (%)			
Spontaneous	23.3	30.7	0.57
Invasive ventilation	72.2	65.3	
Noninvasive ventilation	4.4	4.0	
Blood transfusion (%)	42.9	26.7	0.03

ICU = intensive care unit; MODS = multiple organ dysfunction score; APACHE II score = acute physiology and chronic health evaluation score; SOFA = sequential organ failure assessment; hemoglobin values represent the mean ± standard deviation of all daily values over the 28-day period after transfusion.

**Table 3.** Multivariate analysis for hospital mortality

Variables	P	OR	95% CI	
Blood transfusion	0.011	2.67	1.254	5.686
Male	0.013	2.49	1.209	5.137
Chronic disease	0.029			
Cardiovascular disease	0.003	6.71	1.912	23.578
Respiratory disease	0.087	3.96	0.821	19.128
Renal disease	0.49	1.69	0.369	7.719
Hepatic disease	0.40	2.83	0.251	31.919
Immunodeficient disease	0.021	6.23	1.321	29.379
Baseline MODS	0.009	1.18	1.043	1.336
Clinical patients	0.10	1.83	0.887	3.812

MODS = multiple organ dysfunction score; OR = odds ratio; CI = confidence interval.

**Table 4.** Comparison between transfusion and non-transfusion groups

Variables	All patients	Transfusion (n = 59)	Non-transfusion (n = 108)	P
Age	66.7 ? 13.8	64.2 ? 15.4	68.1 ? 12.8	0.08
Male (%)	58.7	64.4	55.6	0.28
Female (%)	41.3	35.6	44.4	
APACHE II	28.9 ? 6.5	29.5 ? 6.9	28.5 ? 6.2	0.32
Baseline SOFA	6.3 ? 2.8	6.4 ? 2.4	6.2 ? 2.9	0.55
Baseline MODS	7.9 ? 3.0	8.0 ? 2.8	7.8 ? 3.1	0.63
Mean daily hemoglobin total (g/dl)	9.7 ? 1.9	8.7 ? 1.7	9.9 ? 1.8	< 0.01
Origin (%)				
Ward	44.3	42.4	45.4	0.86
Surgery	39.5	40.7	38.9	
Emergency department	15.6	16.9	14.8	
Other hospital	0.6	0.0	0.9	
Previous disease (%)				
Cardiovascular	55.4	57.6	54.2	0.21
Renal	11,4	13.6	10.3	
Immunodeficient	10.2	11.9	9.3	
Respiratory	9.6	3.4	13.1	
Hepatic	2.4	0.0	3.7	
Absent	10.8	13.6	9.3	
Ventilation mode (%)				
Spontaneous	26.5	22.0	29.0	0.06
Invasive ventilation	69.3	78.0	64.5	
Noninvasive ventilation	4.2	0.0	6.5	
Surgery (%)				
Elective	55.1	48.3	59.2	0.35
Emergency	44.9	51.7	40.8	
ICU stay (days)	10.0 (5.0-19.0)	20 (3.0-83.0)	8.0 (3.0-63.0)	< 0.01
Hospital stay (days)	17.0 (10.0-30.0)	24(3.0-140)	14 (3.0-80.0)	0.002
ICU mortality (%)	45.2	66.1	48.6	0.03
Hospital mortality (%)	54.8	88.1	69.4	0.007

ICU = intensive care unit; MODS = multiple organ dysfunction score; APACHE II score = acute physiology and chronic health evaluation score; SOFA = sequential organ failure assessment; hemoglobin values represent the mean ± standard deviation of all daily values over the 28-day period after transfusion; values including a range in brackets represent the median (with 25-75 percentiles).

## DISCUSSION

Anemia has the consequence of decreasing the ability to supply oxygen to tissues and may increase the risks of morbidity and mortality.^13^^,^^14^^,^^15^ It is thus associated with increased length of hospital stay and worse organ dysfunction scores.^15^

Moreover, treatment with blood transfusions is associated with nosocomial infections, in a direct relationship with the number of transfusion units.^6^^,^^16^ Other complications include acute lung injury related to transfusion (TRALI).^17^^,^^18^

Anemia can be treated or tolerated. Several studies have demonstrated that a restrictive strategy in relation to blood transfusion is safe and effective.^8^^,^^19^ Hajjar et al. recently presented a randomized controlled clinical trial among patients undergoing cardiac surgery with cardiopulmonary bypass, comparing a restrictive strategy with a liberal strategy, using a hematocrit cutoff point ³ 24%. They found that restrictive therapy was safe in that population.^19^ However, the evidence for benefits through this strategy is scarce. The study by Herbert et al. demonstrated better outcomes only for patients with less severe conditions (APACHE II < 20) and for younger patient (< 55 years).^8^ Indeed, just like in all the other studies,^20^^,^^21^ no benefits were demonstrated, despite the strategy. However, currently in many intensive care units, it is common practice to define anemia as a hemoglobin level of less than 7 g/dl, with the aim of then maintaining the level between 7 and 9 g/dl.^1^^,^^4^^,^^19^

Our study was conducted among medical and surgical patients who remained in the intensive care unit for more than 72 hours. This length-of-stay criterion was used because we believe that such patients would be more likely develop severe anemia. The data in the literature show that, regardless of this cutoff point, about 95% of such patients already have hemoglobin levels below normal.^2^ Our results showed that 40.1% of these patients received blood transfusions, and this rate is consistent with other studies.^9^^,^^15^ In most cases (77%), the indication for blood transfusion was due to hemoglobin levels of less than 7.0 g/dl. Although the hospital mortality rate found among our patients seemed to be high (54.8%), it was consistent with the severity of these cases, as reflected in high APACHE II scores on admission (28.9 ? 6.5). Blood transfusion was found to be an independent risk factor for mortality (P = 0.01).

The comparison between patients who received transfusions and those who did not showed that although the patients’ ages, gender, APACHE II, baseline SOFA, MODS, underlying disease and origin were not statistically different between the groups, the hospital and intensive care unit lengths of stay and the mortality rates in the hospital and intensive care unit were higher in the group of patients who received transfusions (Table 4). In addition, there was no improvement in SOFA on day 28.

Some authors have argued that transfusion is a marker for disease severity^22^ and have even asked whether the high mortality was due to anemia or transfusion. However, although anemia is associated with high mortality,^13^^,^^14^^,^^15^ anemia correction through blood transfusion does not necessarily mean a reduction in mortality. Several studies have demonstrated increased mortality associated with blood transfusion,^7^^,^^8^ and there are also some studies that showed increased mortality risk proportional to the number of units received.^23^^,^^24^^,^^25^ Moreover, despite the presence of lower hemoglobin levels in the transfusion group, the hemoglobin level was not an independent risk factor for hospital mortality in the multivariate analysis. We believe that transfusion is harmful and is therefore associated with high mortality.

In addition, the development of adverse health consequences from anemia partly depends on each patient’s ability to compensate for these changes.^26^ Anemia is better tolerated in younger patients without comorbidities such as coronary, cerebrovascular or respiratory diseases,^26^ and thus, such patients require fewer blood transfusions.^27^

Furthermore, studies among human volunteers have found that isovolemic hemodilution occurred within the hemoglobin concentration range ? 5.0 g/dl, but that this did not result in evident anaerobic metabolism.^9^ Studies on Jehovah’s Witness patients have shown that survival is possible, even at lower hemoglobin levels. In one case report on a patient whose hemoglobin level was 1.8 g/dl, no major complications were found and the hospital outcome was satisfactory.^28^

Hence, determining who and when to transfuse, and what would be the best trigger for transfusion, is the difficult task that clinicians currently face.^29^ Simple evaluation of the hemoglobin level seems to be insufficient for making decisions regarding blood transfusion, because of the high mortality rate within the transfusion group. Our result showed that patients who had received a blood transfusion because of tissue hypoperfusion had a lower mortality rate than seen among patients who had received a blood transfusion because their hemoglobin level was lower than 7.0 g/dl. One study has shown that using the perfusion parameter gave rise to better accuracy of blood transfusion indications, thereby resulting in better outcomes for patients.^30^

Indeed, decisions regarding transfusions should be individualized. This means that through taking into considerations patients’ ages, previous diseases and perfusion parameters, the complications relating to transfusions can be minimized.^30^^,^^31^

Another important issue is the number of units to be transfused. Ideally, transfusion should be undertaken unit by unit.^16^^,^^32^^,^^33^ On average, the effect from each unit of red blood cells can vary from patient to patient according to age, height, blood storage time and presence of comorbidities such as renal failure and splenectomy.^34^

Some controlling factors in this study need to be considered. The red blood cell storage time was not evaluated, although some studies have suggested that red cells with longer storage time are less efficient for improving the oxygen supply and that the risk of pneumonia may increase by 1% for each day that the red cells are stored.^35^^,^^36^ Another factor that was not studied was the process of leukoreduction in blood, which could influence the evolution of transfused patients.^37^^,^^38^

In addition, the design of this study did not assess mortality in relation to time of occurrence of the transfusion. The transfusions might have occurred later, when the patient was in a worse clinical condition. In recent study, surgical patients transfused with leukoreduction showed increased mortality associated with blood transfusion at a later time.^29^

Finally, it is important to highlight that this was an observational study, and not a randomized study. Therefore, further studies are necessary to clarify the data found so far.

## CONCLUSION

Critically ill patients develop multifactorial anemia, which is progressive with the length of intensive care unit stay. A restrictive transfusion strategy has proven to be safe, but even using this strategy, it is associated with increased morbidity and mortality among some patients, like critically ill patients, besides hypoperfusion associated to low hemoglobin level appears to be an important indicator to be taken into consideration to decide for blood transfusion.
